# Telemedicine Workplace Environments: Designing for Success

**DOI:** 10.3390/healthcare2010115

**Published:** 2014-02-24

**Authors:** Elizabeth A. Krupinski

**Affiliations:** Department of Medical Imaging & Arizona Telemedicine Program, University of Arizona, 1609 N Warren Bldg 211, Tucson, AZ 85724, USA; E-Mail: krupinski@radiology.arizona.edu; Tel.: +1-520-626-4498; Fax: +1-520-626-4376

**Keywords:** telemedicine, environment, workspace, human factors

## Abstract

When designing a facility for telemedicine, there are several things to consider from a human factors point of view, as well as from a practicality point of view. Although the future practice of telemedicine is likely to be more of a mobile-based practice and centered more in the home than it is now, it is still very important to consider ways to optimize the design of clinic-based telemedicine facilities. This is true on both ends of a consultation—where the patient is and where the consultant is. On the patient side, the first thing to realize is that most telemedicine clinics are not going to be newly designed and built. In all likelihood they will be existing rooms converted to telemedicine clinic rooms. Quite often the former room will not even have been used for clinical purposes, but may have simply been a storage area cleared out for telemedicine use. Therefore, design is often a challenge but there are a few basic principles that can be followed to create a workable clinical space. This paper will review some of the basic human factors principles to take into account when designing a working telemedicine environment.

## 1. Clinical Room Design

Although the future practice of telemedicine is likely to be more of a mobile-based practice [1,2,3,4,5,6,7,8] and centered more in the home than it is now [9,10], it is still very important to consider ways to optimize the design of clinic-based telemedicine facilities. Whether you are building a new facility or renovating an existing space, there are a number of important factors to keep in mind. In many cases renovated facilities may not even have been used originally for clinical purposes, but may have simply been a storage area cleaned up for telemedicine use. Under these circumstances, designing a clinical space for telemedicine consultations can be a challenge, but by remaining flexible and adhering to some core principles, a workable and appropriate space for patient encounters can be created [11].

An early consideration is the cohort of patients that are likely to be seen or what types of consultations are going to take place [12,13,14]. For example, a clinic that is dedicated to geriatric patients needs to account for their vision, hearing and mobility challenges in the room design [15]; while a clinic dedicated to pediatrics needs to perhaps include toys or books to entertain them while they are waiting for the consultation to begin; and a telesurgery clinic needs to position cameras and other equipment around the wide array of existing surgical equipment and tools [12].

### 1.1. Physical Considerations

The physical location of the room is important—a room stationed near the out-patient check-in wing of a hospital or clinic will likely have a wide variety of cases referred to telemedicine simply by proximity and familiarity; but if situated in the pediatric wing, it will attract such cases, and if situated in a general practice it may suffer because of the difficulty in sending patients to a dedicated wing. An easy solution is to have a dedicated telemedicine room with portable equipment that is easy to deploy and transport to the patient. With dermatology, for example, this is quite easy as the key piece of equipment is a digital camera and perhaps some dedicated task lighting. It may be impractical to transport a cardiology patient to the telemedicine clinic, but there are portable EKG devices that can be taken to the patient. Even real-time (RT) services are becoming increasingly portable using tablet and SmartPhone devices and roaming robots. Portable devices are extremely useful for example with elderly and disabled patients as it alleviates the burden and time of having to walk from one area of the hospital to another.

Once the location is determined, it is useful to plan the layout in advance—measure it and draw a floor plan. The design should include features such as where the doors and windows will go, where HVAC (heating, ventilation, are conditioning) vents are, where electricity and plumbing fixtures are, where telecommunications lines are, where lighting fixtures are, and so on. At this stage it is useful to include those who are going to use the facility as they may have insights non-users may lack. It might even help to act out clinical scenarios so equipment and furniture needs and locations can be anticipated and space accounted for. For example, a teleneurologist may want to evaluate patient gait and ability to navigate between locations by having them walk from one end of a room to the other [16]. For this RT videoconferencing (VTC) equipment has to be situated properly at both the referring and consulting sites and must be flexible enough to move the camera as the patient walks. The referring tele-clinic must be long enough to accommodate this walk, must be free of obstacles to avoid tripping or falling, and have enough room to accommodate walkers and other assistive devices.

### 1.2. Other Aspects to Consider

All of the other telemedicine equipment (e.g., electronic stethoscopes, digital cameras, docking stations) needs to be strategically placed as well. The room should not be crowded and lots of wires hanging loose or running along the floor is definitely not recommended. Patients with mobility and stability problems or using a cane, walker or wheelchair should not have to worry about tripping on loose wires, bumping into furniture/equipment and possibly injuring themselves.

Most telemedicine clinics will conduct both RT and SF (store-forward) consultations so need to be designed to accommodate both types of equipment. The room design will vary of course, but at least it must accommodate all the equipment and two people (patient and healthcare provider/telepresenter) comfortably. In all likelihood, a desk with a computer with fax or scanner will serve as the site for transferring images and other case data for the teleconsultant. All the required equipment should be organized and within easy reaching distance for those preparing and conducting the teleconsultations. Standard ergonomic concerns about monitor height, monitor distance, mouse placement and task lighting should be observed [17].

An ambient environment also needs to be considered. For example, in some areas, fine dirt or dust blowing in from the outside can ruin equipment and cause problems for patients (e.g., in a tele-COPD clinic where patients already have respiratory problems). Dust filters for the air system could help. Heat control is another issue as telemedicine rooms often have lots of electronic equipment that may generate significant amounts of extra heat. Ill and stressed patients may react poorly if placed in a small hot room for even short amounts of time. The use of fans to keep the environment cool would work well and dehumidifiers are useful in humid environments.

### 1.3. Making Things Work Smoothly

Telemedicine is clearly not television, but it is useful to think of it in this way especially with regard to lighting. With the variety of consultation protocols and options available, a single lighting system may not suffice. When there are no patients, standard office lighting for computer environments can be used [17]; but brighter lights may be needed when a patient is present. The lighting should be adjusted as appropriate during exams or when images are being acquired.

If practice guidelines are available for performing teleconsultations in a given subspecialty, they should be closely followed when technical specifications are provided. The American Telemedicine Association (ATA) has a number of very useful practice guidelines [18]. For example, the teledermatology guidelines recommend that when acquiring digital photos, the room should be well lit (150 ft candles) using light sources as close to white light as possible, and fluorescent day-light or full-spectrum bulbs should be used instead of incandescent bulbs [19]. For RT teleconsultants the best lighting is 300–500 lux and should be angled away from the participants so you may need to buy fixtures on a pole to direct the light appropriately.

In a video-based encounter, everyone needs to clearly see each other and look as close to “normal” as possible with respect to skin tone, *etc*. since these are often diagnostic clues. Again, the patient population is important—light levels for older adults generally need to be about 50% higher than those for younger people (this aids their color perception as well). It is probably easier to establish a trusting relationship if the patient is comfortable and is not straining to see things. For example, part of a teleconsultancy may involve showing a patient the label on a medication package, the color of the pills, or some other information, and it is important that the patient be able to see the information clearly so they can better understand it.

Believe it or not, the color of the walls and floors is critical as well. Light blue is often recommended, but only one or two walls not the whole room—you do not want the color temperature to affect the appearance of the skin (*i.e.*, people should not look bluer than they would naturally) [13]. Flat latex blue paint should be used rather than gloss or semi-gloss to avoid glare and reflections. This is often a problem since most hospitals prefer glossy paints as they are easier to clean, but there is a legitimate reason for requesting matte or flat paints.

Clutter is another thing to avoid. Teleconsult participants should be able to focus on each other without distracting details or objects in the background. Cameras and monitors are important. Stationary cameras should be directed towards the patient from about 5 feet away to capture the most information possible (*i.e.*, head plus full body shots) and placed on top of the monitor used for viewing. This is easier with dedicated telemedicine carts than it is with desktop or mobile (e.g., tablet, phone) units since these devices typically are placed or held much closer to the users and provide basically head shots. The limitations of these mobile devices should be recognized and considered before using them and, if wider views are needed, it may require another person in the room to hold the device farther away. While large monitor screens (≥50”) are useful in the clinic setting, smaller monitors also work well. For the most part, resolution is adequate, especially with HD (high-definition) devices. HD cameras and displays are readily available at a reasonable cost, so if possible they should be used.

### 1.4. Display and Audio Considerations

Traditional desktop displays and mobile devices are very useful for reviewing SF data. If radiographic images are going to be reviewed, then adequate spatial and contrast resolution is required. It is not necessary to use a medical-grade radiology display [20], but it should be properly calibrated for reading radiographic images (preferably to the level of DICOM GSDF—Digital Imaging and Communications in Medicine Grayscale Standard Display Function) [21,22]. While there are some standards regarding the acquisition of images for some of the many telemedicine applications (e.g., teledermatology [19]), guidance on calibration of color displays for medical images is fragmented without consensus regarding what type of calibration should be performed even within a given clinical specialty. A single validated color display calibration protocol is not in place for color image applications in medicine. There are however some very easy methods to at least standardize the appearance of color images on digital displays and they should be used to insure some degree of consistency [23].

Sound is very important as well in RT teleconsultations. Speaker and microphone capabilities and placement are important and need to accommodate all types of users. For example, some patients (e.g., elderly) are often soft-spoken or have trouble projecting so the microphone should be placed closer to them than other patients. With mobile devices it is quite easy to move the device closer to the speaker, but if the same device serves as the camera it obviously can be a problem. The speakers may also have to be placed closer or have the volume turned up so as to compensate for hearing loss/difficulties. Again, with mobile devices adjusting for sound by moving the device impacts the video component. In all consultations the patient should be asked at the beginning of the teleconsultancy if they can comfortably see and hear the teleclinician, and adjustments made if necessary.

## 2. Non-Clinic-Based Teleconsultations

Many of the considerations detailed above apply when the patient and/or healthcare provider is outside of the clinical environment (e.g., home, office, school). Clearly it is not possible to change the wall color or buy new lighting for a patient (although the provider can if it is going to be his/her regular location), but it is possible to ask them to try to optimize the environment by holding the tele-visit in a room without a lot of background clutter, with good light levels that have some adjustability, to have the camera face a wall instead of a window and so on. It is also useful on both sides to find a location that avoids a lot of competing sounds (e.g., family members, tv, radio, *etc.*). This is not only important for reducing noise distractions, but if these noise sources are identified and minimized, it is more likely that privacy will be assured which is critical. In general it may be useful for healthcare providers to refer to the ATA’s “Practice Guidelines for Video-Based Online mental Health Services”, as this document specifically addresses providing consultations using alternative/mobile devices in non-clinical environments [24].

### 2.1. Privacy Issues

In general, it is recommended that the rooms the provider and patient are in should be fairly comparable to standard services rooms. Again, ensuring privacy so clinical discussions cannot be overheard by others is critical. When other people are present, both the provider and patient should be made aware of the other person(s) and agree to their presence. Cameras (as high a quality as possible) should be on a secure, stable platform to avoid wobbling and shaking. This is pretty easy with desktop displays, but tablets and phones may need to be secured somehow. The cameras should be placed at eye level with the face clearly visible to the other person.

In clinical environments, privacy and security measures are usually set in place by the IT (information technology) team as a function of the more global hospital, state and federal regulations surrounding these issues. In non-clinical environments these issues are a little harder to deal with, so precautions need to be taken. Some of the more important things to consider include the following points [24]. Video software platforms that include social media functions that notify users when anyone on a contact list logs on should not be used. Platforms with free video chat applications and the ability to create video chat rooms should be disabled. The computers should have current antivirus software (the latest security patches and updates applied to the operating system) and a personal firewall installed if possible (at least on the providers side). A backup plan (e.g., phone) is useful to have in case there are technology issues or breakdowns. Screen-in-screen options (a.k.a., picture-in-a-picture) are useful and are available in most video-conferencing software packages.

### 2.2. Mobile Environments

SmartPhones represent the new frontier in telemedicine. There have been a number of studies on the use of mobile devices and phones for teleradiology, telepathology and other image-based specialties [25,26,27,28,29,30,31,32,33,34,35,36,37], but these are not especially relevant to RT teleconsultations between patients and healthcare providers using these types of devices (this does not include the use of mobile apps as this does not really involve consulting or the use of cameras in a clinical encounter). There need to be more studies done to validate the reliability and validity of using mobile devices for teleconsulting applications and perhaps guidelines established for safe and effective use.

One important feature of the devices approved by the FDA for the mobile teleradiology applications is an interactive contrast test in which a small part of the screen is a slightly different shade than the rest of the screen. If the physician can identify and tap this portion of the screen, then the lighting conditions will not interfere with the physician’s ability to discern subtle differences in contrast. It is not simply a question of whether the physician can see the target—he/she actually needs to locate and identify it—a built in safety measure. Being able to detect low contrast targets is obviously critical to viewing radiographic images, but it seems likely that similar tools can readily be developed for other remote data and image viewing applications (store-forward and real-time) that could help insure that participants are in conditions that are amenable to viewing images, data and each other if engaged in a real-time teleconsultancy. The FDA has yet to step into this area of telemedicine, but they are actively looking at, for one example, telepathology and the potential need to regulate how monitors are calibrated for the display of color medical images. Whether this would extend to other applications such as teleophthalmology and teledermatology is an open question.

## 3. Conclusions

Telemedicine is changing and will continue to change—not only in the way it is practiced but in the environments in which it is practiced. It is important to manage these environments from a variety of perspectives and taking all stakeholders into account [38,39,40]. It is impossible to predict what types of devices we will be using to communicate and send data to one other, but it is clear that no matter in what direction we head, there will be more mobile solutions, and patients and healthcare providers will be connecting with each other more often, and in different ways, than at present. The environments within which healthcare interactions take place are going to be as varied as the providers and patients, but the fundamental concerns and principles about creating spaces that facilitate clear, open and caring communication should guide our efforts to successfully—and with proper integrity, security and respect for privacy—reach out and connect with those who need care: anywhere, anytime.
